# Weightlifting derivatives vs. plyometric exercises: Effects on unloaded and loaded vertical jumps and sprint performance

**DOI:** 10.1371/journal.pone.0274962

**Published:** 2022-09-22

**Authors:** Ricardo Berton, Demostenys David da Silva, Marcel Lopes dos Santos, Claudio Machado Pinto e Silva, Valmor Tricoli

**Affiliations:** 1 School of Physical Education and Sport, University of São Paulo, São Paulo, Brazil; 2 School of Kinesiology, Applied Health, and Recreation, Oklahoma State University, Stillwater, OK, United States of America; Universidade Federal de Mato Grosso do Sul, BRAZIL

## Abstract

The aim of this study was to compare the effects of weightlifting derivatives (WL) and plyometric exercises (PLYO) on unloaded and loaded vertical jumps and sprint performance. Initially, 45 resistance-trained men underwent a 4-week WL learning period. Then, the participants were randomly assigned to 1 of 3 groups (WL (n = 15), PLYO (n = 15), and control group (CG) (n = 15)) and followed a training period of 8 weeks. The WL group performed exercises to stimulate the entire force-velocity profile, while the PLYO group performed exercises with an emphasis in vertical- and horizontal-oriented. The CG did not perform any exercise. Pre- and post-training assessments included peak power output (PPO) and jump height (JH) in the squat jump (SJ), countermovement jump (CMJ), CMJ with 60% and 80% of the body mass (CMJ60% and CMJ80%, respectively), and mean sprinting speeds over 5, 10, 20, and 30 m distances. From pre- to post-training, PLYO significantly increased (p≤0.05) PPO and JH in the SJ, PPO during CMJ, and PPO and JH in the CMJ60%; however, no significant changes were observed in JH during CMJ, and PPO and JH in the CMJ80%. For WL and CG, no significant changes were observed in the unloaded and loaded vertical jumps variables. PLYO also resulted in significant improvements (p≤0.05) for 5, 10, and 20 m sprint speeds, but not for 30 m. For WL and CG, no significant changes were observed for all sprint speeds. In conclusion, these data demonstrate that PLYO was more effective than a technically-oriented WL program to improve unloaded and loaded vertical jumps and sprint performance.

## Introduction

The ability to generate high forces and power during unloaded and loaded motor tasks (e.g., vertical jumps and sprint, and opponents’ projection in combat sports, respectively) is crucial for athletic performance [1–3]. For this reason, strength and power development has been a primary goal of several training programs. To accomplish this goal, strength and conditioning coaches have implemented weightlifting exercises and their derivatives (WL) [4–7] and also plyometric exercises (PLYO) [7, 8], in addition to traditional resistance training (free-weight exercises such as squat at high intensity).

The use of WL and PLYO in sports training programs [7] is related to their putative benefits. Researchers have shown that both training methods promote significant improvements in unloaded and loaded vertical jumps and sprint performance [9–17]. Not only the effectiveness of WL and PLYO are widely recognized, but the comparison between them is also well-documented, especially for unloaded vertical jumps (e.g., squat jump (SJ) and countermovement jump (CMJ)) [9, 12, 14, 15, 18]. Briefly, researchers have described superior increases in the peak power output (PPO) during SJ and CMJ when implementing WL compared to PLYO-based programs [9, 12, 14]. On the other hand, some researchers have described similar performance improvement between both training methods for jump height (JH) when assessed during the SJ and CMJ [9, 10, 19, 20]. From a practical standpoint, this information is essential for strength and conditioning coaches to make a better decision when selecting exercises to be included in a training program. However, while SJ and CMJ have been constantly compared, other important motor tasks remain poorly investigated.

The loaded vertical jump may represent an athlete’s ability to apply PPO during loaded actions including opponent’s projection in combat sports and physical contact activities in American football. To the best of the authors’ knowledge, no study has compared the effects of the WL and PLYO on PPO during loaded vertical jumps. Nevertheless, it is plausible to suggest that the heavy loads used during WL would elicit greater improvements on PPO compared to PLYO [16]. The PPO during loaded vertical jumps is mainly affected by the ability to produce force [21–23]. In turn, the ability to produce force is substantially enhanced when training with heavy loads [21, 24, 25]. In this perspective, WL may be more advantageous, as exercises without the catch phase (e.g., mid-thigh clean pull) can be performed with heavier loads and therefore, provide an adequate stimulus to increase maximum strength and therefore, force production ability [13]. In contrast to this advantage, during PLYO, loading is commonly restricted to the body mass (lighter loads) [8]. This fact may contribute to lower improvements on maximum strength [26]. Therefore, based on the exercise’s appropriate choice (i.e., without catch phase and with heavy loads), WL may be a better option for enhancing PPO during loaded vertical jump.

The sprint performance is another motor task poorly compared between WL and PLYO. Tricoli et al. [15] observed greater improvement for WL compared with PLYO at 10 m, while in two other studies, the researchers reported similar performances between training methods at 5 and 20 m [14, 15]. Although these results indicate a superiority for WL or at least, a similar performance between training methods, it is important to note the absence of horizontal-oriented exercises for the groups that performed PLYO [14, 15]. The possibility of performing horizontal-oriented exercises (e.g., horizontal jumps) is a specific advantage of PLYO to maximize horizontal force production and potentially, to induce greater improvements in sprint performance at short distances (10 m) when compared to vertical-oriented exercises [11, 27]. Following this rationale, when horizontal jumps exercises are used in PLYO based training protocols, a greater sprint performance may be expected for PLYO when compared to WL.

Therefore, the purpose of this study was to compare the effects of the WL and PLYO-based programs on unloaded and loaded vertical jumps and sprint performance. It was hypothesized that (a) WL would induce greater improvements on PPO during unloaded vertical jumps, while both training methods would improve JH similarly, (b) WL would induce higher PPO during loaded vertical jump, and (c) PLYO would induce higher sprint performance.

## Materials and methods

### Experimental design

Initially, all participants underwent a 4-week weightlifting derivatives learning period. After the learning period, the initial testing sessions were undertaken. In the first and second sessions, participants performed both vertical jump conditions (i.e., unloaded and loaded), in the third and fourth sessions, the 30 m sprint was tested, and in the fifth and sixth sessions, the 1-repetition maximum (1RM) test in the half-squat exercise was applied (just for the characterization of the sample). Every test was performed twice, in two separate sessions, to verify the reliability. After the initial testing sessions, the participants were randomly assigned to 1 of 3 possible groups (WL, PLYO, or control group), and then, they initiated an 8-week training period. Five days after the last training session, participants were submitted to the post-training tests. The first session was intended for the unloaded and loaded vertical jumps and the second session for the 30 m sprint. Intervals of 72-96h were allowed between testing sessions.

### Participants

Forty-five males participated in the study (Table 1). All participants were engaged in resistance training for at least 1 year and they had a 1RM to body mass ratio in the half-squat exercise ≥1.5 kg•kg^-1^. However, the participants had no experience in the weightlifting exercises (snatch and clean and jerk) and their derivatives. All participants gave their informed consent before enrollment in the study. The study was conducted according to the Declaration of Helsinki and the University’s Research Ethics Committee approved the experimental protocol.

**Table 1:** Participants’ characteristics at pre-training.

Groups	N	Age	Height	BM	1RM/BM (kg•kg^-1^)
		(years)	(m)	(kg)	
WL	15	24.1 ± 4.2	1.75 ± 0.1	77.7 ± 8.8	2.12 ± 0.4
PLYO	15	23.9 ± 4.7	1.74 ± 0.1	77.2 ± 9.8	2.16 ± 0.3
CG	15	24.3 ± 3.2	1.76 ± 0.1	83.6 ± 9.1	2.10 ± 0.2

Data are presented as mean ± standard deviation. N = number of participants, BM = body mass, 1RM = half-squat 1-repetition maximum test, WL = weightlifting derivatives, PLYO = plyometric exercises, CG = control group.

### Vertical jumps

The participants performed a general warm-up on a cycle ergometer for 5 minutes at 20 km•h^-1^. Two-minutes after, they executed a specific warm-up composed of 4 submaximal SJ and CMJ attempts. After a 3-minute interval, they performed 5 maximal SJ and CMJ [1]. It was allowed an interval of 10–15 seconds between jumps, and a 3-minute interval between SJ and CMJ [1].

In the SJ, the participants remained in a static position with a 90° knee angle for ∼2-s before jumping. A goniometer was used to determine knee flexion angle. To ensure correct positioning in all attempts, it was used 2 wooden sticks connected by an elastic band (see S1 Fig). A trial was excluded if any countermovement was observed. The countermovement was defined as a ground reaction force of 10 N below the system weight. In the CMJ, the participants were instructed to perform a countermovement using a self-selected depth, followed immediately by the lower limbs joints complete extension. Moreover, SJ and CMJ were performed with hands on the hips.

Ten minutes after the last CMJ, the participants performed the CMJ with 60% (CMJ60%) and 80% (CMJ80%) of body mass. These trials were performed on a Smith machine. Participants underwent a specific warm-up composed of 2 submaximal CMJ with 45% and 65% of body mass. Three-minutes after, they performed 3 maximal CMJ60% and 2 maximal CMJ80%. It was allowed an interval of 10–15 seconds between jumps, and a 3-minute interval was granted between CMJ60% and CMJ80%. Participants were instructed to perform a countermovement using a self-selected depth, followed immediately by the lower limbs joints complete extension [28]. However, during all jumps, their hands had to hold the barbell at all times, and in case the barbell lost contact with the shoulders, the trial was considered invalid [1].

All vertical jump tests were performed on a force plate (AccuPower, AMTI, Watertown, MA, EUA). Data from the force plate were collected at a sample frequency of 1000 Hz. The force plate was connected to a computer and ground reaction force data was analyzed via AccuPower 3.0 software (AMTI, Watertown, MA, USA). For all vertical jumps, the participants were weighed on the force plate, standing as still as possible for 3 seconds. For CMJ60% and CMJ80%, the participants were weighed with the external load on their shoulders to determine the system mass (body + barbell) [28]. The initiation of the trials was defined as the first instant when ground reaction force was 10 N above (SJ) or below (CMJ, CMJ60%, and CMJ80%) the system weight [29]. The end of trials (end of the concentric phase) for all vertical jumps was defined as the instant at which ground reaction force was 5 N below system weight [29]. For CMJ, CMJ60%, and CMJ80%, the initiation of the concentric phase was considered as the instant at which the center of mass velocity exceeded 0.01 m.s^-1^ [30]. After collecting the vertical ground reaction force during jumps, the impulse-momentum approach was used to calculate the velocity of the center of mass of the system [29, 31]. Then, maximum values of force and velocity acquired during the concentric phase of the vertical jumps were used to calculate the system mass PPO [28]. The JH was determined by the take-off velocity [32]. The average of all valid attempts was used for subsequent statistical analyses.

### Sprint test

The sprint test was carried out on a 30 m straight line. The participants performed a general warm-up composed of 5 minutes of jogging. After that, a specific warm-up composed of 1 submaximal attempt at 50%, 70%, and 90% of maximal estimated effort was performed over 30m [13]. Three-minutes after, the participants performed the sprint test consisted of 3 maximal attempts. A 3-minute interval was allowed between attempts [13]. Only the standing start was allowed [33].

The MySprint application (Apple Inc., USA) was used to measure the sprint test variables [34]. The sprint test was filmed with an iPad Pro (Apple Inc, Cupertino, CA, USA) with a built-in 240 fps high-speed camera at a quality of 720p [34]. The camera was positioned on a tripod in the sagittal plane at the 15 m marker, and 10 m from the track. Six markers were positioned according to the MySprint’s recommendations to measure the time at 5, 10, 15, 20, 25, and 30 m, respectively. The time on each distance was determined when the participant’s hip was exactly aligned with each parallax marker [34]. Although MySprint provides many variables, to test the hypothesis of the present study only the sprinting speeds at 0–5 (5m), 0–10 (10m), 0–20 (20m), and 0–30 m (30m) were analyzed. For statistical analysis, it was used the average values from all valid attempts.

### Learning period

All participants underwent a 4-week learning period with WL exercises (twice a week in non-consecutive days). The learning protocol was composed of the following exercises: (1) extension of the hip and knee, (2) extension of the hip and knee, 1-second pause, and extension of the ankle, (3) extension of the hip and knee, 1-second pause, extension of the ankle, 1-second pause, and clean grip upright row, (4) clean grip upright row followed immediately by the catch phase, (5) mid-thigh clean pull, (6) high pull from the knee, and (7) power clean from the knee. Except for exercises 4 and 5 which started from mid-thigh, all other exercises were initiated with the barbell just above the top edge of the patella (adjusted by wood blocks). For the exercises 1, 2, and 3, participants performed the hip and/or knee extension in the same pattern of the initial movement of the high pull from the knee and power clean from the knee.

Participants performed 3 sets of 6 repetitions for all exercises, with 60-second intervals between sets and exercises. In the first and second weeks, they performed the exercises with a standard Olympic barbell (20 kg) and in the last two weeks, one 5 kg weight plate was added on each side [35]. To maximize learning, verbal feedback was provided in the rest intervals between sets for all exercises [36].

### Training period

WL and PLYO training programs were performed during 8 weeks with 2 sessions per week in non-consecutive days, while the CG did not undergo any training. For WL and PLYO, a criterion of 90% compliance was established. Each group followed training protocols with specific sets and repetitions (Table 2); however, a rest interval of 2 minutes was adopted between sets and exercises for both training groups.

**Table 2:** Training programs.

Groups	Exercises	Weeks 1–4 (sets x repetitions)	Weeks 5–8 (sets x repetitions)
WL	High pull from the knee	4 x 6	5 x 6
	Power clean from the knee	4 x 4	5 x 4
	Mid-thigh clean pull	3 x 3	3 x 3 + 1 x 2
PLYO	Bounce drop jump	5 x 5	6 x 5
	Double-leg hurdle hops	5 x 5	6 x 5
	Horizontal jumps	4 x 5	5 x 5

WL = weightlifting derivatives, PLYO = plyometric exercises.

The WL group performed the following exercises: high pull from the knee, power clean from the knee, and mid-thigh clean pull. These exercises were chosen due to their ability to stimulate the entire force-velocity profile [5]. All exercises were performed from adjustable blocks and participants were instructed to execute the concentric phase of each repetition as fast as possible. The heaviest load that could be lifted without compromising the appropriate exercise technique was used [16]. Therefore, the load prescription was not based on the percentage of the 1RM test. All loads were adjusted for all exercises over the 8 weeks of the training period. The load progression data for the power clean from the knee are presented in S2 Fig. In the first session, the researcher determined the first load based on the information of the learning period of each participant.

The PLYO group performed the following exercises: bounce drop jump, double-leg hurdle hops, and horizontal jumps. These exercises were chosen due to their ability to improve vertical- and horizontal-oriented sports tasks [27]. In all exercises, the participants were instructed to perform each repetition as fast as possible during the concentric phase and to minimize contact time with the ground. The drop-box height was determined by the reactive strength index (jump flight time divided by ground contact time). Each participant performed in the pre-training assessment 4 different drop height tests (0.20, 0.30, 0.40, and 0.50 m). The drop height that allowed the participant to achieve the highest reactive strength index was used throughout the training program [37]. The median drop height was 0.30 m (2, 7, 5, and 1 participant used drop heights of 0.2, 0.3, 0.4, and 0.5 m, respectively). For the double-leg hurdle hops, the distance between hurdles was 1.5 m, with a minimum height at 0.6 m. All exercises were executed in hard surface (gymnasium floor).

### Statistical analyses

Statistical analyses were performed using IBM SPSS software version 22 (IBM, New York, NY, USA). Data normality and variance homogeneity were assessed through the Shapiro-Wilk’s and Levene’s tests, respectively. Reliability between pre-training testing sessions was assessed using the coefficient of variation (CV) [38]. To compare pre-training values (participants’ characteristics, SJ, CMJ, CMJ60%, CMJ80%, 5, 10, 20, and 30m) between groups, a one way analysis of variance was performed. As there were no significant differences between groups at pre-training values (p>0.05), a mixed model was applied for each dependent variable. Group (WL, PLYO, and CG) and time (pre- and post-training) were defined as fixed factors and participants as a random factor. When a significant F value was obtained, a Tukey post hoc test was utilized. The significant level was set at *p*≤0.05. Data are presented as mean ± standard deviation (SD). Finally, the estimated mean and SD delta changes from each group were used to calculate between-group effect sizes (ES) (Hedge’s *g*, Eq 1) and the associated 95% confidence intervals (CI) [39, 40]. The criteria for the qualitative inferences of the ES were: ≤0.19 (trivial), 0.20–0.59 (small), 0.60–1.19 (moderate), and 1.20–1.99 (large) [41]. However, if the CI overlapped thresholds for positive and negative values, the effect was considered unclear [40].


$$\mathrm{Hedge}’s\;g=\frac{M2-M1}{\mathrm{SD}*\mathrm{pooled}}\cdot (1‐\frac{3}{4(n1+n2-2)‐1})$$
**Eq 1:** Hedge’s g with bias correction.

## Results

All data were normally distributed and presented similar variance. Regarding reliability, the CVs for PPO were: 2.34%, 1.95%, 2.20%, and 3.63% in the SJ, CMJ, CMJ60%, and CMJ80%, respectively. For JH, the CVs were: 2.98%, 3.09%, 4.09%, and 7.03% in the SJ, CMJ, CMJ60%, and CMJ80%, respectively. In the sprint test, the CVs were: 1.97%, 0.98%, 0.97%, and 0.93% for 5, 10, 20, and 30 m, respectively.

### Unloaded vertical jumps

Pre- to post-training absolute values are shown in Table 3. Following the 8-week training period, there was a significant group-time interaction for PPO in the SJ (*p* = 0.0001). PPO in the SJ increased significantly in the PLYO group at post-training (*p* = 0.0001, +7.0%), while no significant changes were observed in the WL group (*p* = 0.11, +2.5%) and CG (*p* = 0.99, -0.37%). There was also a significant group-time interaction for JH in the SJ (*p* = 0.0005), and again, only PLYO group significantly increased (*p* = 0.0006, +7.8%) at post-training, while no significant changes were observed in the WL group (*p* = 0.32, +3.1%) and CG (*p* = 0.69, -2.1%). For PPO in the CMJ, there was a significant group-time interaction (*p* = 0.0006) with a significant increase at post-training in the PLYO group (*p* = 0.0003, +5.2%), and no significant changes in the WL group (*p* = 0.48, +1.8%) and CG (*p* = 0.83, -1.1%). However, for JH in the CMJ, no significant group-time interaction was observed (*p* = 0.19). Only descriptively, the percentage changes for JH in the CMJ were: +3.5% for the PLYO group, +0.1% for the WL group, and -3.3% for CG. Table 3 also presents the ES comparisons between-groups. Briefly, WL and PLYO groups presented favorable ES (moderate to large) for almost all SJ and CMJ variables compared to the CG (excepted between WL and CG for JH in the CMJ (ES = unclear)). Moreover, PLYO group also presented favorable ES (moderate to large) for PPO and JH in the SJ and PPO in the CMJ compared to the WL. Between WL and PLYO groups, the JH in the CMJ was considered unclear.

**Table 3:** Changes in the unloaded and loaded vertical jumps from pre- to post-training for the weightlifting derivatives (WL), plyometric exercises (PLYO), and control group (CG).

	WL		PLYO		CG		Effect size, (CI) and [qualitative inference]		
	Pre	Post	Pre	Post	Pre	Post	WL vs. PLYO	WL vs. CG	PLYO vs. GC
**SJ**									
PPO (W•kg^-1^)	54.1 ± 4.1	55.5 ± 4.2	51.1 ± 5.7	54.6 ± 5.5*	52.4 ± 5.8	52.2 ± 6.6	-1.21 (-2.04–0.44) [Large]	0.83 (0.08 1.61) [Moderate]	1.81 (0.95 2.74) [Large]
JH (cm)	34.4 ± 3.7	35.5 ± 3.9	32.7 ± 3.9	35.2 ± 4.2*	34.4 ± 5.4	33.8 ± 5.4	-0.79 (-1.56–0.04) [Moderate]	1.01 (0.26 1.81) [Moderate]	1.48 (0.67 2.37) [Large]
**CMJ**									
PPO (W•kg^-1^)	54.2 ± 4.5	55.2 ± 5.0	52.7 ± 6.1	55.4 ± 5.9*	54.2 ± 5.2	53.5 ± 5.1	-0.93 (-1.71–0.19) [Moderate]	0.73 (0.01 1.48) [Moderate]	1.46 (0.67 2.30) [Large]
JH (cm)	39.4 ± 5.2	39.4 ± 5.5	38.5 ± 4.9	39.9 ± 5.2	39.7 ± 5.7	38.7 ± 5.1	-0.72 (-1.48 0.02) [Unclear]	0.43 (-0.28 1.16) [Unclear]	1.00 (0.25 1.78) [Moderate]
**CMJ60%**									
PPO (W•kg^-1^)	47.8 ± 5.3	48.8 ± 5.3	45.9 ± 5.7	48.4 ± 5.8*	48.0 ± 4.2	46.9 ± 4.5	-0.47 (-1.21 0.23) [Unclear]	0.93 (0.16 1.74) [Moderate]	1.44 (0.63 2.32) [Large]
JH (cm)	16.8 ± 3.4	17.6 ± 3.6	15.7 ± 3.0	17.0 ± 3.4*	17.2 ± 2.9	16.8 ± 2.9	-0.49 (-1.23 0.22) [Unclear]	0.95 (0.18 1.75) [Moderate]	1.35 (0.54 2.21) [Large]
**CMJ80%**									
PPO (W•kg^-1^)	47.5 ± 5.7	48.1 ± 5.3	46.0 ± 6.27	48.2 ± 6.4	47.1 ± 3.8	46.6 ± 4.2	-0.24 (-0.97 0.48) [Unclear]	0.56 (-0.18 1.33) [Unclear]	0.81 (0.04 1.61) [Moderate]
JH (cm)	13.5 ± 3.2	13.8 ± 2.9	12.6 ± 3.2	13.6 ± 3.1	13.3 ± 2.3	13.3 ± 2.5	-0.14 (-0.87 0.57) [Unclear]	0.46 (-0.27 1.22) [Unclear]	0.61 (-0.15 1.39) [Unclear]

Values pre- and post-training are presented as mean ± standard deviation. CI = 95% confidence interval, SJ = squat jump, CMJ = countermovement jump, CMJ60% = countermovement jump with 60% of body mass, CMJ80% = countermovement jump with 80% of body mass, PPO = peak power output, JH = jump height.

* Significant difference from pre-training (p ≤ 0.05).

**Fig 1:**
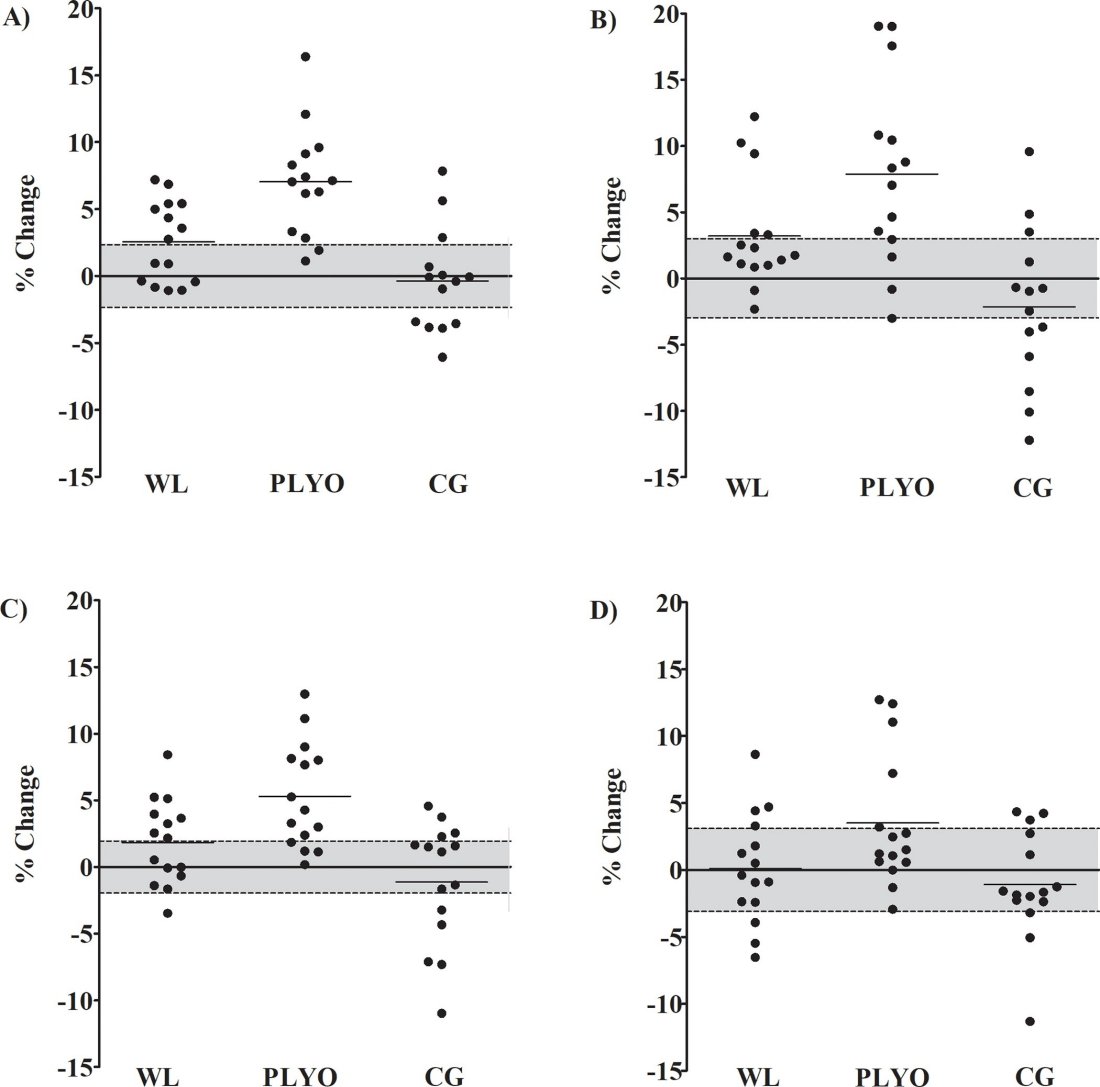
Unloaded vertical jumps mean (horizontal lines ^____^) and individual (black circles) percentage changes from pre- to post-training. Grey area represents the coefficient of variation (%). A) Peak power output in the squat jump, B) jump height in the squat jump, C) peak power output in the countermovement jump, and D) jump height in the countermovement jump. WL = weightlifting derivatives; PLYO = plyometric exercises; CG = control group.

The individual percentage changes are presented in Fig 1. Descriptively, PPO was increased in the SJ above the CV in 53.3%, 85.7%, and 21.4% of the sample for the WL, PLYO, and CG groups, respectively. For JH in the SJ, it was observed an increased above the CV in 33.3%, 71.4%, and 21.4% of the sample for the WL, PLYO, and CG groups, respectively. For PPO in the CMJ, it was observed an increased above the CV in 53.3%, 73.3%, and 26.6% of the sample for the WL, PLYO, and CG groups, respectively. On the other hand, JH was increased in the CMJ above the CV in 26.6%, 33.3%, and 20% of the sample for the WL, PLYO, and CG groups, respectively.

### Loaded vertical jumps

Pre- to post-training absolute values are shown in Table 3. For PPO in the CMJ60%, a significant group-time interaction was observed (*p* = 0.002). The PLYO group demonstrated a significant increase at post-training (*p* = 0.01, +5.6%), while no significant changes were observed in the WL group (*p* = 0.69, +2.3%) and CG (*p* = 0.45, -2.9%). For JH in the CMJ60%, a significant group-time interaction was also observed (*p* = 0.002). Jump height increased in the PLYO group at post-training (*p* = 0.01, +8.9%), while no significant changes were observed in the WL group (*p* = 0.56, +4.1%) and CG (*p* = 0.52, -4.0%). On the other hand, for PPO and JH in the CMJ80% there were no significant group-time interactions (*p* = 0.12 and *p* = 0.31, respectively). Descriptively, the percentage changes for PPO in the CMJ80% were: +3.4%, +1.6%, and -1.7% for the PLYO, WL, and CG, respectively. For JH in the CMJ80%, the percentage changes were: +5.0%, +3.2%, and -1.5% for the PLYO, WL, and CG, respectively. Effect size comparisons between-groups are also presented in Table 3. Briefly, WL and PLYO groups presented favorable ES (moderate to large) for all CMJ60% variables compared to the CG. However, unclear ES was observed between training groups and CG for almost all CMJ80% variables (except between PLYO and CG for PPO (ES = moderate)). Between WL and PLYO groups, the PPO and JH in the CMJ60% and CMJ80% were considered unclear.

**Fig 2:**
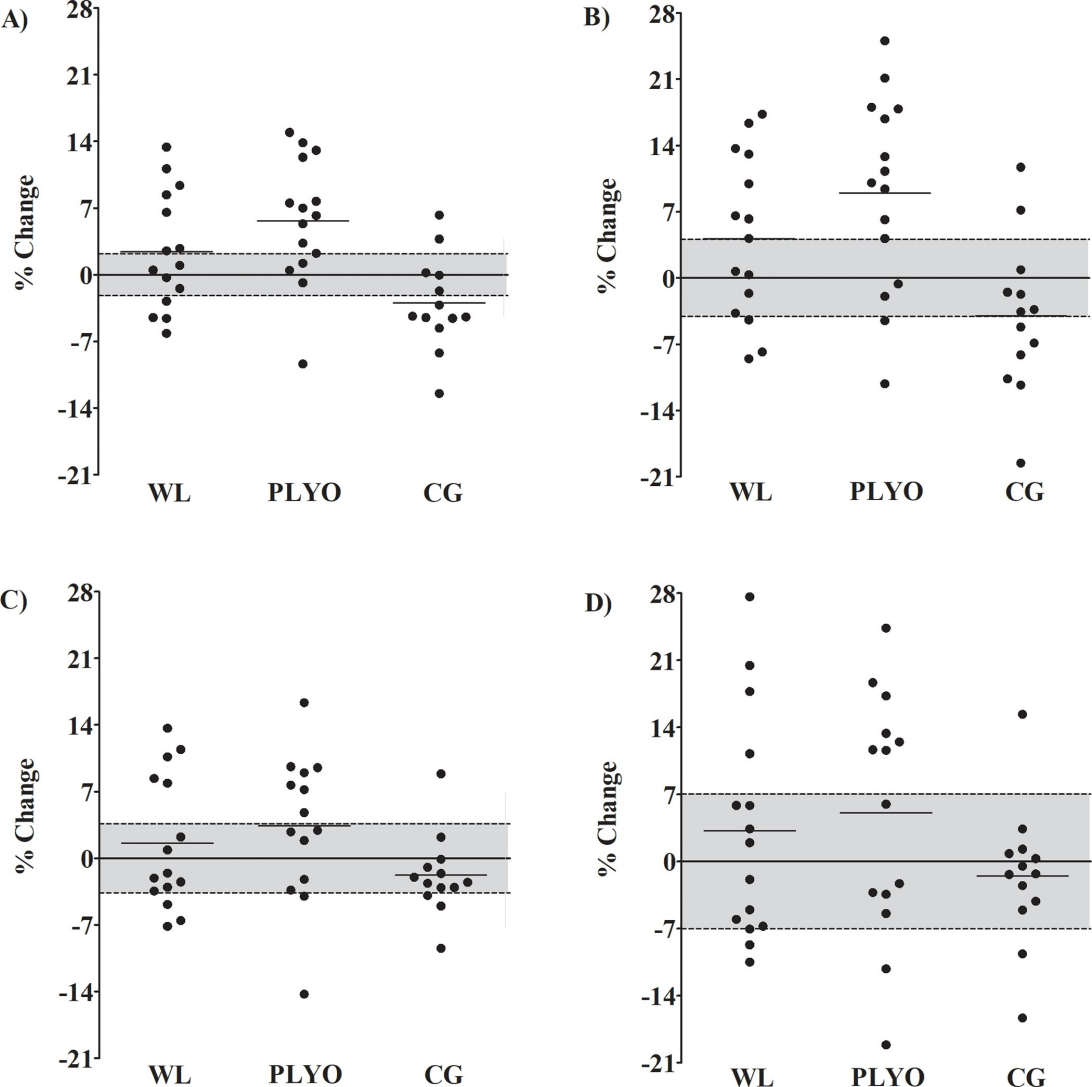
Loaded vertical jumps mean (horizontal lines ^____^) and individual (black circles) percentage changes from pre- to post-training. Grey area represents the coefficient of variation (%). A) Peak power output in the countermovement jump with 60% of the body mass, B) jump height in the countermovement jump with 60% of the body mass, C) peak power output in the countermovement jump with 80% of the body mass, and D) jump height in the countermovement jump with 80% of the body mass. WL = weightlifting derivatives; PLYO = plyometric exercises; CG = control group.

The individual percentage changes are presented in Fig 2. Descriptively, PPO was increased in the CMJ60% above the CV in 46.6%, 66.6%, and 15.3% of the sample for the WL, PLYO, and CG groups, respectively. For JH in the CMJ60%, it was observed increases above the CV in 53.3%, 73.3%, and 15.3% of the sample for the WL, PLYO, and CG groups, respectively. For PPO in the CMJ80%, it was observed increases above the CV in 33.3%, 50%, and 7.6% of the sample for the WL, PLYO, and CG groups, respectively. On the other hand, JH was increased in the CMJ80% above the CV in 26.6%, 50%, and 7.6% of the sample for the WL, PLYO, and CG groups, respectively.

### Sprinting speed

Pre- to post-training absolute values are shown in Table 4. For 5 m, a significant group-time interaction was observed (*p* = 0.02). There was an increase for 5 m at post-training in the PLYO group (*p* = 0.01, +2.8%) and no significant changes in the WL group (*p* = 0.38, +1.4%) and CG (*p* = 0.98, -0.4%). For 10 m, a significant group-time interaction was also observed (*p* = 0.001). There was an increase for 10 m at post-training in the PLYO group (*p* = 0.0002, +2.6%), while no significant changes were observed in WL group (*p* = 0.35 +1.0%) and CG (*p* = 0.96, -0.4%). There was also a significant group-time interaction for 20 m (*p* = 0.003), and again, PLYO group showed a significant increase at post-training (*p* = 0.002, +1.6%), while no significant changes were observed in WL group (*p* = 0.98, +0.2%) and CG (*p* = 0.94, -0.3%). For 30 m, there was no significant group-time interaction (*p* = 0.12). Only descriptively, the percentage changes for 30 m were: +1.2%, -0.2%, and -0.5% for the PLYO, WL, and CG, respectively. ES between-groups comparisons are shown in Table 4. WL group presented a favorable ES (moderate) for 5 and 10 m when compared to CG. However, an unclear ES was observed between WL and CG for 20 and 30 m. PLYO group presented a favorable ES (moderate to large) in all sprint speeds when compared to CG. Between training methods, the PLYO group presented an unclear ES for 5 and 10 m when compared to WL; however, a favorable ES (moderate) for 20 and 30 m.

**Table 4:** Changes in sprint speeds from pre- to post-training for the weightlifting derivatives (WL), plyometric exercises (PLYO), and control group (CG).

	WL		PLYO		CG		Effect size, (CI) and [qualitative inference]		
	Pre	Post	Pre	Post	Pre	Post	WL vs. PLYO	WL vs. CG	PLYO vs. GC
5 m (m•s^-1^)	3.86 ± 0.26	3.93 ± 0.25	3.76 ± 0.23	3.86 ± 0.19*	3.87 ± 0.20	3.85 ± 0.17	-0.45 (-1.20 0.29) [Unclear]	0.88 (0.09 1.71) [Moderate]	1.04 (0.21 1.86) [Moderate]
10 m (m•s^-1^)	4.82 ± 0.26	4.88 ± 0.25	4.69 ± 0.23	4.81 ± 0.20*	4.82 ± 0.18	4.81 ± 0.17	-0.72 (-1.50 0.02) [Unclear]	1.09 (0.28 1.95) [Moderate]	1.35 (0.51 2.24) [Large]
20 m (m•s^-1^)	5.84 ± 0.28	5.87 ± 0.27	5.71 ± 0.33	5.80 ± 0.28*	5.84 ± 0.22	5.83 ± 0.25	-0.96 (-1.77–0.19) [Moderate]	0.44 (-0.32 1.24) [Unclear]	1.29 (0.47 2.18) [Large]
30 m (m•s^-1^)	6.39 ± 0.31	6.40 ± 0.30	6.24 ± 0.40	6.33 ± 0.35	6.38 ± 0.29	6.36 ± 0.32	-0.94 (-1.75–0.17) [Moderate]	0.23 (-0.53 1.01) [Unclear]	1.08 (0.27 1.94) [Moderate]

Values pre- and post-training are presented as mean ± standard deviation. CI = 95% confidence interval, 5 m = sprinting speed at 0–5 m, 10 m = sprinting speed at 0–10 m, 20 m = sprinting speed at 0–20 m, 30 m = sprinting speed at 0–30 m.

* Significant difference from pre-training (p ≤ 0.05).

**Fig 3:**
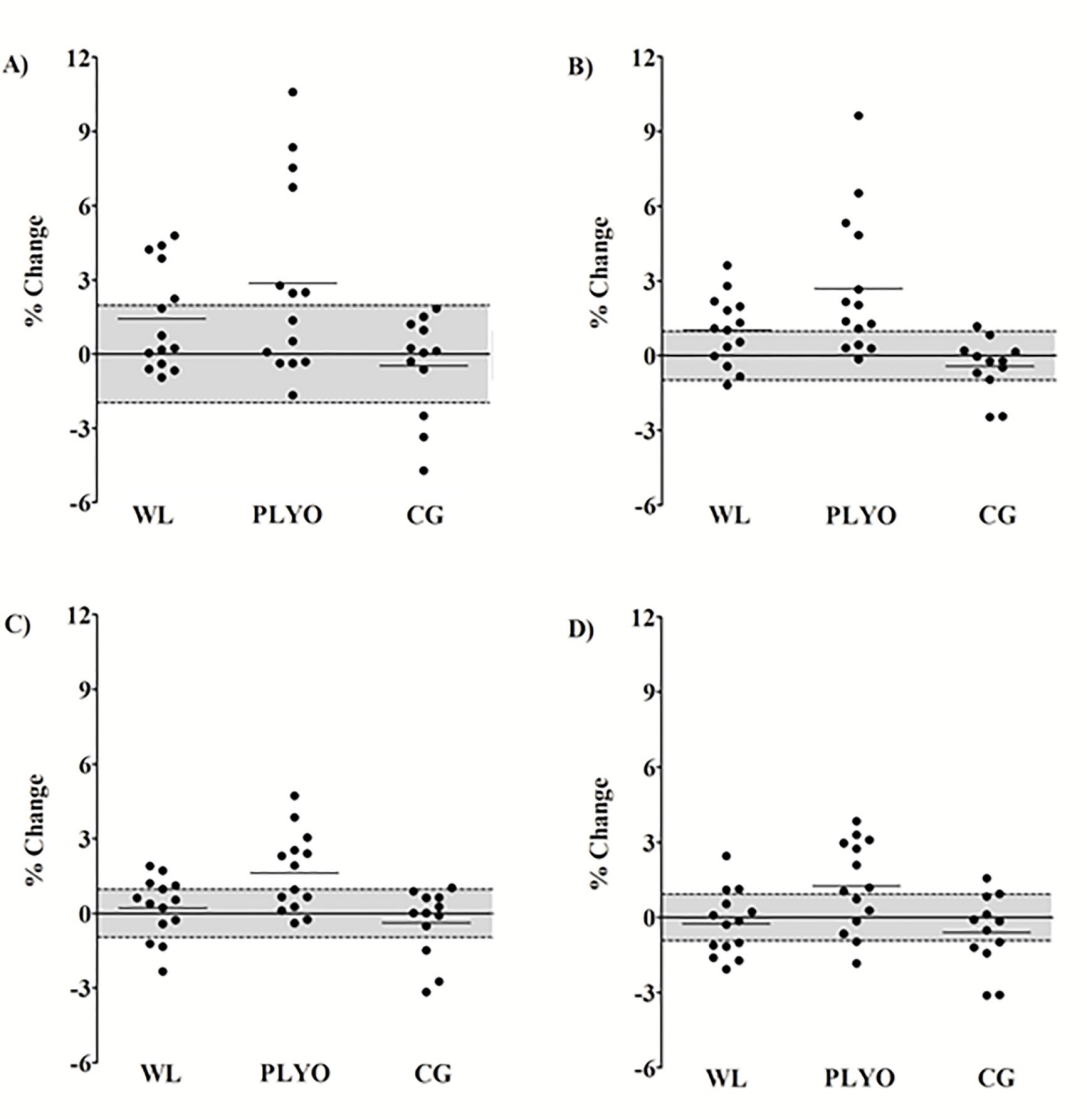
Sprint speed mean (horizontal lines ^____^) and individual (black circles) percentage changes from pre- to post-training. Grey area represents the coefficient of variation (%). A) sprinting speed 0–5 m, B) sprinting speed 0–10 m, C) sprinting speed 0–20 m, and D) sprinting speed 0–30 m. WL = weightlifting derivatives; PLYO = plyometric exercises; CG = control group.

The individual percentage changes are presented in Fig 3. Descriptively, 5 m increased above of the CV in 35.7%, 50%, and 0% of the sample of the WL, PLYO, and CG groups, respectively. For 10 m, there was an increase above the CV in 57.1%, 71.4%, and 8.3% of the sample for the WL, PLYO, and CG groups, respectively. For 20 m, there was an increase above the CV in 35.7%, 57.1%, and 8.3% of the sample for the WL, PLYO, and CG groups, respectively. Finally, for 30 m there was an increase above the CV in 21.4%, 57.1%, and 16.6% of the sample for the WL, PLYO, and CG groups, respectively.

## Discussion

The purpose of this study was to compare the effects of WL and PLYO on unloaded and loaded vertical jumps and sprint performances. As result, our data demonstrate that PLYO was more effective than a technically-oriented WL program to improve unloaded and loaded vertical jumps and sprint performance.

For unloaded vertical jumps, both training methods were more effective than CG (Table 3). Between training methods, the PLYO group induced greater improvements than the WL group for PPO and JH in the SJ and PPO in the CMJ (Table 3). In addition to the significant changes occurred only in the PLYO and favorable ES compared to WL group (Table 3), the individual percentage changes above the CV also occurred more frequently for the PLYO group (Fig 1). Taken together, these results allow to suggest a superiority of the PLYO compared to the WL group for SJ (PPO and JH) and CMJ (PPO).

The superiority of the PLYO group on unloaded vertical jump performance variables found in the present study is not in accordance with the literature. Some researchers have shown that the use of WL-based training programs promotes greater PPO augmentation during SJ and CMJ compared to PLYO [9, 12, 14]. In addition, similar responses for SJ and CMJ height have been observed between training methods [9, 10, 19, 20]. The reason for these conflicting results is not totally known; although, the divergent results might be explained by the load prescription and training protocols. The WL exercises in our study were prescribed based on the heaviest load that could be lifted with proper technique. Although this method of load prescription is simple and practical for some real sports training programs, its limitation should not be discarded. It is possible to suggest that due to the absence of an accurate load prescription strategy such as the percentage of the 1RM test, the WL group did not improve the performance of the unloaded vertical jumps. In this sense, future studies should investigate the influence of different WL exercises load prescription strategies on unloaded vertical jumps performance.

Another hypothesis for the divergent results of the unloaded vertical jumps is related to the training protocols. It is possible to suggest that the inclusion of resistance exercises into PLYO and/or WL protocols might have contributed to these different responses. For example, Hawkins et al. [12] included resistance exercises (e.g., front and back squat) only in the WL protocol. The inclusion of these exercises might have contributed to an additional effect on PPO and JH in the SJ and CMJ and, consequently, resulted in greater improvements in the WL group compared to the PLYO. Other researchers also compared the effectiveness of WL and PLYO with the resistance exercises included, although for both training methods [9, 14, 15]. The inclusion of resistance exercises in both training programs eliminates any possible external advantage of a training method over the other, and still allows greater ecological validity as resistance exercises are usually implemented in these training programs. However, it should be recognized that the inclusion of resistance exercises precludes the verification of the intrinsic effects of WL and PLYO [16]. Moreover, it is not entirely clear whether resistance exercises may maximize WL effects in greater magnitude than PLYO. Therefore, it should not be ruled out the possibility that the results of the present study are not in line with previous studies due to the absence of resistance exercises in our training protocols.

Regarding the loaded vertical jumps, both training methods were more effective than CG for PPO and JH in the CMJ60% (Table 3). Between training groups, the PLYO also resulted in superior improvements for PPO and JH for CMJ60% when compared to the WL group (Table 3). Besides the significant changes, the superiority of the PLYO group compared to the WL group may be represented by the higher frequency of individual percentage changes above the CV (Fig 2). On the other hand, the changes induced by the PLYO and WL groups in the CMJ80% were not greater than the CV (Fig 2). Thus, the results observed in the CMJ80% variables may be considered only as measurement errors and not necessarily an adaptation induced by training.

The greater effectiveness of the PLYO in the loaded vertical jump (i.e., CMJ60%) might be related to the characteristics of the loaded jumps, load prescription, and the exercises used in the present study. Contrary to regular CMJ, loaded vertical jumps are usually performed with greater countermovement depth [42]; fact observed in the present study (CMJ = 0.38 m CMJ60% = 0.64 m, CMJ80% = 0.69 m). This characteristic is relevant as the mid-thigh clean pull, the main WL that could affect loaded vertical jumps [5], was performed with a small range of motion (start position from mid-thigh height). Thus, it is possible to suggest that the increase in force production was limited only to a small range of motion and it did not result in a significant impact in PPO and JH during loaded vertical jumps. Corroborating this logic, only the study by Helland et al. [16] was able to show an increase in PPO production in loaded vertical jumps after a WL protocol. However, differently from the present study, they used exercises with greater ranges of motion (i.e., exercises performed from the floor, such as the clean and power clean). According to the present study results and the argumentation previously mentioned, WL starting from the knee position, although less complex (due to the absence of the transition phase), does not seem to improve loaded vertical jumps. Additional studies should be conducted to investigate the influence of WL’s range of motion on loaded vertical jumps performance. Not only the range of motion, but also the load prescription of the WL exercises should be highlighted. As mentioned earlier in this discussion, the WL exercises in our study were prescribed based on the heaviest load that could be lifted with proper technique. Although the mid-thigh clean pull was performed from 3 repetitions by set (likely close to subjects’ 1RM) (Table 2), the absence of a more accurate load prescription (based on 1RM test) may not have allowed an adequate stimulus of the strength-speed zone. As consequence, the WL group may not have maximized the performance in the loaded vertical jumps.

While in the WL group, the range of motion and load prescription might explain the results found in the loaded vertical jumps, for the PLYO group, the reasons for the improvement in CMJ60% are not entirely clear. However, it is possible to suggest that the significant increases in the CMJ60% (PPO and JH) induced by the PLYO training might be related to small increases in muscular strength and the similar movement pattern between PLYO and CMJ60%. Although usually performed only with the body mass [8], PLYO may induce small but significant improvements in strength-related measures such as maximal isometric and dynamic strength, and 5 maximum repetitions [26, 43]. The positive changes of the strength-related measures are relevant, as they indicate that PLYO may optimize an important factor for performance in the loaded vertical jumps, the ability to produce maximum force [22–24]. Moreover, the similarity between PLYO and CMJ60% should be mentioned. In contrast to WL, PLYO and CMJ60% share a similar movement pattern (i.e., vertical jump). This similarity may have facilitated the transfer of the small increases in muscular strength acquired by PLYO to CMJ60% performance. Therefore, the present study results indicate that PLYO may positively affect PPO and JH in loaded vertical jumps. However, the improvement induced by PLYO seems to have a limited effect, as significant changes were observed for CMJ60% but not for CMJ80%.

Regarding the sprint performance, both training methods were also more effective than CG (Table 4), and again, the PLYO group promoted superior effects compared to the WL group in all sprint speeds (5, 10, 20, and 30 m) (Table 4). Not only by significant changes observed in the PLYO group but also by favorable ES compared to WL group (Table 4), the PLYO group superiority may also be represented by the higher frequency of individual percentage changes above the CV for all sprint speeds (Fig 3). The results of the present study do not corroborate with previous research. For instance, Tricoli et al. [15] showed greater improvements at 10 m sprint speed for the WL compared with the PLYO group and similar results at 30 m between training methods. Teo et al. [14] also showed similar improvements between WL and PLYO; however, at 5 and 20 m. These divergent findings may be explained by the inclusion of the horizontal jumps only in our PLYO protocol. The use of horizontal jumps increases the ability to produce horizontal force and; consequently, induces greater improvements at 10 m sprint speed than the vertical-oriented exercises [11, 27]. The application of horizontal jumps in our PLYO protocol may have contributed to the greater improvement in sprint performance (i.e., 5 and 10 m) compared to the vertical-oriented exercises used by the WL group. In addition to better results in short distances, PLYO group also provided greater improvements at 20 and 30 m sprint speeds compared to WL group. The advantage achieved at 20 and 30 m sprint speeds may be more related to the vertical-oriented exercises on PLYO protocol [27]. However, even to a lesser magnitude, the use of horizontal jumps may also contribute to improved 20 m sprint speed [27]. Thereby, it seems reasonable to infer that the horizontal and vertical jumps in our PLYO protocol maximized the performance at 5, 10, 20, and 30 m sprint speeds.

Finally, some limitations need to be acknowledged. First, the countermovement depth was self-selected in the CMJ, CMJ60%, and CMJ80%. The non-standardization of the countermovement depth may influence the push-off phase duration and; consequently, affect PPO and JH [28]. Nevertheless, the countermovement depth during CMJ, CMJ60%, and CMJ80% performed in the pre- and post-training tests was measured and no significant differences were identified (*p*>0.05) between and within groups. Second, the WL exercises were initiated from a concentric contraction. Although this may be seen as beneficial for the present sample due to the use of less complex exercises, WL exercises that initiate with an eccentric contraction (hang pull and countermovement shrug) may be more advantageous to stimulate the stretch-shortening cycle and PPO [44, 45]. Third, due to the different characteristics between WL and PLYO, it was not possible to equalize total training volume and intensity. In order to reduce these limitations, the present study equalized the total number of repetitions executed in the vertical-oriented exercises (880 repetitions for each group) and the percentage increment in the number of repetitions was similar between groups after the fifth week of training (WL = 19.6% and PLYO = 16.6%). Fourth, the present study also used a small sample size, with experience in resistance training, but no experience in the weightlifting exercises and their derivatives. Even though the present study used a similar or larger sample size than other studies that compared WL vs. PLYO (range 7–15 participants) [46], a 4-week WL learning period and 2 of the 3 exercises without the catch phase, a possible influence of small sample size and WL learning on dependent variables should not be ruled out. Lastly, the WL group’s training program was designed to stimulate the entire force-velocity profile. However, due to the absence of a more precise method to quantify the load prescription, such as percentage of the 1RM, force and velocity may not have been maximized. Although this fact is a limitation, it is important to note that the implementation of the 1RM test for the load prescription may be challenging in certain real sports training programs (e.g., very time-consuming/labor-intensive) [47]. In this sense, the prescription used in the present study may reflect the reality of certain sports training scenarios.

## Conclusion

In conclusion, these data demonstrate that PLYO was more effective than a technically-oriented WL program to improve unloaded and loaded vertical jumps and sprint performance. It should be noted that the results of the present study were acquired in a short-term training program (8-week), with WL load prescriptions not based on the 1RM test, and the sample had experience in resistance training, but not in weightlifting derivatives. Therefore, we advise the use of PLYO instead of WL in situations of short periods of preparation (≤ 8-week), absence of 1RM test for WL, and for practitioners who have no experience in WL.

## Supporting information

S1 Dataset (PDF)

S1 Fig (A) Adjustable wooden structures positioned on each side of the force plate (black arrows = measuring tapes, white arrow = elastic band); (B) example of the adjustment for the squat jump. After using a goniometer to determine the 90° knee flexion angle, the range of motion was demarcated by contact of the gluteus with the elastic band; (C) aerial phase of the squat jump.
(PDF)

S2 Fig The load progression in the WL group was represented by the adjustments performed in the power clean from the knee exercise. (PDF)
