# Supplementary material for: Weightlifting derivatives vs. plyometric exercises: Effects on unloaded and loaded vertical jumps and sprint performance
Source: PLoS One. 2022 Sep 22;17(9):e0274962. doi: 10.1371/journal.pone.0274962 (PMC9499257; doi:10.1371/journal.pone.0274962)
Supplement: S1 Dataset — (PDF) [file pone.0274962.s001.pdf]

	SJ				CMJ			
	jump height		peak power output		jump height		peak power output	
Control group	Pre	Pos	Pre	Pos	Pre	Pos	Pre	Pos
1	39,29	38,32	57,19	57,14	41,49	40,55	57,70	56,91
2	37,11	32,58	52,56	50,76	40,36	35,79	54,15	50,19
3	37,90	37,53	52,04	52,08	42,64	41,80	52,83	54,17
4	29,67	28,58	45,85	45,67	32,75	34,17	47,05	47,80
5	44,39	46,54	63,06	68,00	49,49	50,05	60,52	63,28
6	34,00	37,26	52,98	55,96	44,31	46,18	57,39	58,69
7	33,22	33,64	51,36	51,31	40,38	39,72	56,41	52,40
8	28,06	27,87	45,96	46,28	35,03	34,20	49,00	48,19
9	31,36	31,12	52,46	50,42	38,45	36,51	56,42	53,97
10	36,18	33,09	53,91	51,99	49,28	40,11	62,53	55,67
11	43,93	39,49	64,74	60,82	45,56	44,84	61,49	62,41
12	33,48	32,13	50,53	51,98	35,91	35,46	48,70	50,52
13	30,71	.	52,95	.	35,74	34,60	53,04	53,91
14	32,04	30,15	47,84	46,00	34,51	35,45	49,85	48,25
15	25,00	25,87	43,44	43,02	30,95	32,11	46,46	46,98
Mean	34,42	33,87	52,46	52,24	39,79	38,77	54,23	53,56
DP	5,47	5,49	5,87	6,61	5,74	5,17	5,24	5,15
Weightlifting group	Pre	Pos	Pre	Pos	Pre	Pos	Pre	Pos
16	35,93	36,51	57,81	57,20	47,79	46,66	58,30	60,43
17	35,29	35,78	54,15	56,50	39,96	39,80	57,04	58,27
18	34,28	35,15	50,51	51,90	36,96	36,06	49,17	48,35
19	30,00	30,33	47,14	46,97	31,37	32,85	46,14	48,55
20	34,15	37,36	58,77	58,28	44,61	42,17	54,99	54,95
21	35,23	36,05	52,09	54,90	44,38	43,97	60,44	60,04
22	31,96	35,86	53,31	56,96	40,90	41,11	54,72	55,01
23	37,06	38,31	59,55	60,09	39,52	41,26	56,73	58,57
24	33,63	33,33	52,36	51,80	39,27	36,71	54,86	54,85
25	33,20	33,48	54,43	54,20	35,40	34,01	51,37	49,59
26	29,76	30,06	50,00	51,78	32,06	31,77	48,22	47,55
27	45,04	45,83	61,68	64,76	48,15	52,31	61,28	63,71
28	37,66	38,90	55,62	58,63	40,81	42,15	57,68	60,64
29	31,70	30,97	48,86	52,37	35,85	36,30	49,86	54,05
30	31,61	34,84	55,96	56,49	34,65	35,28	52,50	53,84
Mean	34,43	35,52	54,15	55,52	39,45	39,49	54,22	55,23
DP	3,77	3,94	4,16	4,28	5,20	5,59	4,55	5,06
Plyometric group	Pre	Pos	Pre	Pos	Pre	Pos	Pre	Pos
31	35,03	38,10	59,39	61,07	48,42	49,96	63,38	64,14
32	30,28	33,44	45,19	48,94	38,10	42,94	48,91	55,25
33	32,47	35,17	47,68	51,21	36,10	36,54	49,23	50,84
34	31,84	33,31	47,26	50,23	33,27	34,18	46,02	47,11
35	41,11	42,32	59,69	60,83	45,98	45,38	60,90	61,00
36	34,16	34,71	59,61	60,28	42,13	45,17	61,09	64,29
37	36,31	43,22	56,54	63,37	40,85	45,36	54,69	60,78
38	33,69	33,41	51,45	55,12	39,57	40,54	52,88	57,18
39	30,99	34,34	51,70	54,89	36,28	36,67	51,57	52,16
40	.	.	.	.	35,82	36,03	48,77	52,68
41	28,22	30,20	43,14	47,07	31,46	35,36	43,82	47,76
42	32,53	38,73	50,82	59,15	42,88	43,15	58,56	59,65
43	27,63	32,48	47,51	52,08	38,44	38,44	55,04	56,69
44	37,74	36,60	51,58	53,29	37,98	38,56	52,21	56,21
45	26,99	27,95	43,90	46,99	31,58	30,66	44,40	46,29
Mean	32,78	35,28	51,11	54,61	38,59	39,93	52,76	55,47
DP	3,98	4,24	5,78	5,52	4,90	5,27	6,16	5,90

	CMJ60%					CMJ80%			
	jump height		peak power output			jump height		peak power output	
Control group	Pre	Pos	Pre	Pos		Pre	Pos	Pre	Pos
1	14,17	15,83	45,43	48,28		9,75	9,83	44,73	44,70
2	19,47	15,66	50,41	44,13		15,21	12,72	49,65	44,95
3	18,03	19,32	47,90	49,70		14,33	16,53	45,98	50,06
4	14,18	13,03	41,68	39,84		11,00	10,85	42,08	40,97
5	23,24	22,03	54,14	51,11		17,69	16,95	52,41	50,36
6	19,82	19,99	51,53	51,51		15,12	15,31	50,70	50,21
7	15,16	14,93	45,66	43,56		13,12	13,05	46,90	44,54
8	16,99	16,43	48,08	46,55		12,32	12,16	45,80	44,89
9	13,93	12,35	42,73	39,22		10,66	10,12	40,92	39,88
10	19,91	19,20	52,35	51,47		15,61	16,14	51,48	52,61
11	21,63	19,33	56,06	53,62		16,49	16,54	53,65	52,78
12	15,25	14,99	44,12	44,22		11,96	11,66	44,15	42,80
13	16,56	15,42	49,72	47,48		13,47	12,17	48,75	47,25
14	15,50	.	46,36	.		12,51	.	46,62	.
15	14,27	.	43,90	.		11,58	.	43,75	.
Mean	17,21	16,81	48,01	46,97		13,39	13,39	47,17	46,62
DP	2,99	2,90	4,29	4,56		2,30	2,58	3,82	4,27
Weightlifting group	Pre	Pos	Pre	Pos		Pre	Pos	Pre	Pos
16	17,87	20,79	47,00	52,23		15,20	16,91	48,62	53,80
17	22,07	21,25	56,35	53,76		17,47	18,06	54,53	53,6766
18	12,39	13,16	38,89	39,97		7,83	9,43	36,07	38,92
19	11,88	11,92	41,11	40,51		9,10	8,64	41,64	38,91
20	17,40	20,42	47,30	53,64		14,87	15,74	49,04	53,15
21	21,02	20,09	52,35	50,89		17,31	15,80	52,01	49,48
22	18,41	18,54	48,39	48,24		13,90	14,71	46,78	47,19
23	19,63	19,31	53,43	53,69		16,30	15,15	54,62	52,74
24	16,74	15,43	48,47	46,28		13,02	12,78	47,70	46,52
25	14,97	13,69	46,30	43,45		13,09	11,71	47,67	44,27
26	14,63	15,25	44,75	45,88		12,75	11,98	46,31	45,34
27	21,18	23,29	53,06	56,54		18,22	18,57	54,53	55,74
28	18,63	19,86	54,64	55,17		14,23	13,27	52,13	50,55
29	12,00	13,58	40,03	43,78		9,07	10,68	38,65	43,07
30	13,96	15,88	45,37	49,18		10,63	13,56	43,41	49,34
Mean	16,85	17,50	47,83	48,88		13,53	13,80	47,58	48,18
DP	3,41	3,54	5,37	5,37		3,23	2,99	5,71	5,33
Plyometric group	Pre	Pos	Pre	Pos		Pre	Pos	Pre	Pos
31	22,15	24,23	60,18	60,91		19,01	18,39	60,09	61,22
32	16,08	19,48	43,53	49,57		13,14	16,34	42,71	49,69
33	13,75	14,32	43,62	44,59		11,24	9,98	43,57	41,83
34	12,40	13,80	39,97	42,77		8,88	9,92	39,41	41,29
35	18,04	17,92	49,11	48,69		16,64	15,74	52,68	50,92
36	16,17	18,89	47,93	50,90		13,06	15,32	47,42	51,67
37	15,91	17,51	47,75	51,43		12,56	13,31	47,46	48,77
38	11,71	14,64	40,18	46,19		7,07	.	37,07	.
39	16,14	14,33	46,66	42,28		14,08	11,38	48,55	41,62
40	16,16	15,43	46,44	46,66		12,44	12,01	47,31	46,26
41	14,58	16,45	41,21	44,32		11,50	13,04	40,72	43,65
42	20,41	21,67	53,47	56,33		16,01	17,86	51,97	55,96
43	13,44	15,84	41,89	47,06		11,34	13,45	42,75	46,86
44	17,47	20,63	49,12	55,54		14,75	16,59	51,03	55,88
45	11,10	10,88	38,35	39,62		8,21	8,02	38,59	39,72
Mean	15,70	17,07	45,96	48,46		12,66	13,67	46,09	48,24
DP	3,06	3,48	5,76	5,82		3,21	3,17	6,27	6,45

SPRINT

	5 m		10 m		20 m		30 m	
Control group	Pre	Pos	Pre	Pos	Pre	Pos	Pre	Pos
1	.	.	.	.	.	.	.	.
2	.	.	.	.	.	.	.	.
3	3,589	3,623	4,737	4,736	5,869	5,906	6,497	6,552
4	3,66	3,66	4,57	4,61	5,67	5,72	6,25	6,35
5	3,968	3,978	5,015	5,023	6,163	6,227	6,851	6,859
6	3,792	3,780	4,815	4,792	5,927	5,922	6,547	6,514
7	3,922	3,994	4,829	4,838	5,719	5,690	6,150	6,077
8	3,541	3,543	4,507	4,497	5,544	5,545	6,082	6,077
9	4,249	4,107	4,987	4,865	5,765	5,679	6,186	6,124
10	4,063	3,962	5,040	5,005	6,087	6,126	6,686	6,749
11	4,054	3,862	5,061	4,936	6,199	6,029	6,803	6,593
12	3,925	3,984	4,927	4,985	5,993	5,994	6,597	6,587
13	3,931	3,906	4,796	4,785	5,663	5,677	6,146	6,058
14	3,737	3,782	4,640	4,596	5,587	5,410	6,027	5,839
15	3,828	.	4,780	.	5,679	.	6,131	.
Mean	3,87	3,85	4,82	4,81	5,84	5,83	6,38	6,36
DP	0,20	0,17	0,18	0,17	0,22	0,25	0,29	0,32
Weightlifting group	Pre	Pos	Pre	Pos	Pre	Pos	Pre	Pos
16	4,044	4,200	5,062	5,153	6,125	6,162	6,664	6,654
17	4,006	3,980	4,831	4,790	5,683	5,695	6,129	6,196
18	3,978	3,940	4,879	4,929	5,901	5,972	6,465	6,538
19	3,204	3,206	4,186	4,241	5,223	5,313	5,706	5,846
20	3,727	3,736	4,725	4,751	5,781	5,757	6,360	6,251
21	3,967	3,943	4,889	4,831	5,883	5,745	6,381	6,249
22	4,137	4,144	5,124	5,102	6,208	6,132	6,808	6,729
23	4,017	4,092	5,074	5,173	6,215	6,275	6,829	6,865
24	3,506	3,660	4,471	4,596	5,480	5,542	6,018	6,001
25	3,843	4,027	4,735	4,838	5,735	5,766	6,343	6,272
26	3,713	3,797	4,672	4,723	5,642	5,627	6,212	6,112
27	.	4,029	.	5,048	.	6,177	.	6,807
28	4,125	4,156	5,075	5,074	6,098	6,017	6,628	6,560
29	3,865	4,029	4,884	5,061	5,921	6,033	6,466	6,479
30	3,965	3,949	4,870	4,886	5,852	5,875	6,421	6,426
Mean	3,86	3,93	4,82	4,88	5,84	5,87	6,39	6,40
DP	0,26	0,25	0,26	0,25	0,28	0,27	0,31	0,30
Plyometric group	Pre	Pos	Pre	Pos	Pre	Pos	Pre	Pos
31	3,613	3,885	4,704	4,931	5,854	6,002	6,520	6,598
32	3,966	3,900	4,742	4,793	5,646	5,776	6,145	6,273
33	3,296	3,645	4,268	4,546	5,222	5,468	5,730	5,919
34	3,368	3,650	4,175	4,398	4,906	5,096	5,241	5,396
35	3,86	3,867	4,426	4,852	5,854	5,893	6,470	6,408
36	3,702	3,722	4,685	4,786	5,770	5,945	6,363	6,608
37	3,637	3,626	4,714	4,707	5,833	5,818	6,407	6,397
38	3,759	3,745	4,686	4,706	5,529	5,635	5,944	6,127
39	3,997	4,266	4,973	5,074	6,086	6,062	6,758	6,634
40	3,799	3,894	4,798	4,859	5,806	5,844	6,403	6,421
41	3,729	3,779	4,671	4,735	5,634	5,687	6,151	6,196
42	3,957	4,067	5,049	5,065	6,144	6,151	6,705	6,775
43	3,701	3,792	4,607	4,729	5,590	5,725	6,109	6,277
44	4,148	4,132	5,114	5,128	6,078	6,094	6,612	6,569
45	3,902	.	4,746	.	5,635	.	6,097	.
Mean	3,76	3,86	4,69	4,81	5,71	5,80	6,24	6,33
DP	0,23	0,19	0,26	0,20	0,33	0,28	0,40	0,35
