# Supplementary material for: Weightlifting derivatives vs. plyometric exercises: Effects on unloaded and loaded vertical jumps and sprint performance
Source: PLoS One. 2022 Sep 22;17(9):e0274962. doi: 10.1371/journal.pone.0274962 (PMC9499257; doi:10.1371/journal.pone.0274962)
Supplement: S1 Fig — (A) Adjustable wooden structures positioned on each side of the force plate (black arrows = measuring tapes, white arrow = elastic band); (B) example of the adjustment for the squat jump. After using a goniometer to determine the 90° knee flexion angle, the range of motion was demarcated by contact of the gluteus with the elastic band; (C) aerial phase of the squat jump. (PDF) [file pone.0274962.s002.pdf]

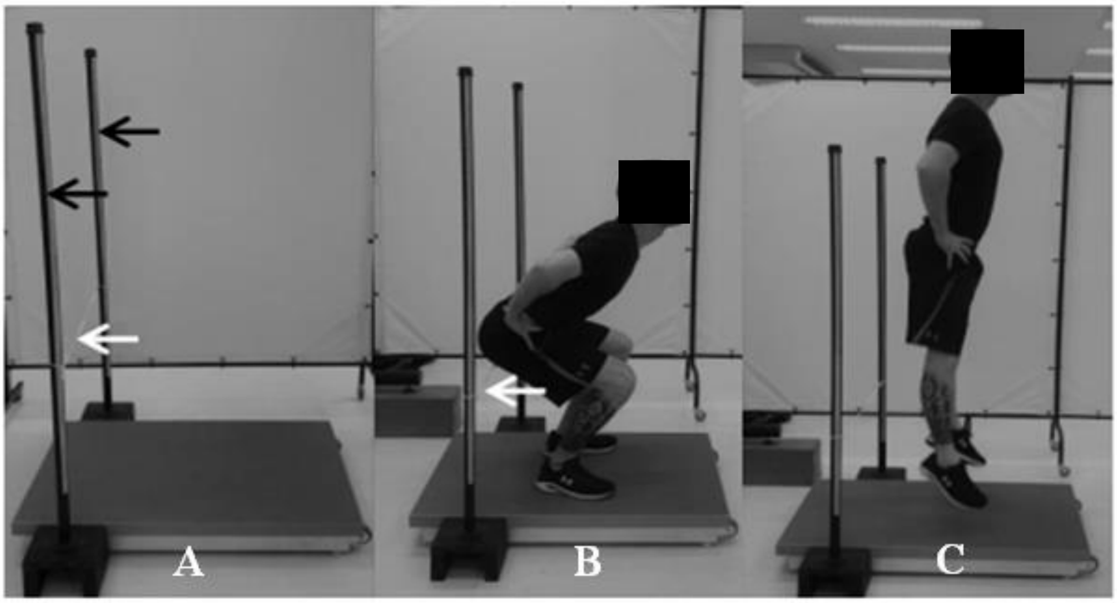

S2 supplemental material. (A) Adjustable wooden structures positioned on each side of the force plate (black arrows = measuring tapes, white arrow = elastic band); (B) example of the adjustment for the squat jump. After using a goniometer to determine the 90º knee flexion angle, the range of motion was demarcated by contact of the gluteus with the elastic band; (C) aerial phase of the squat jump.
