# Supplementary material for: Weightlifting derivatives vs. plyometric exercises: Effects on unloaded and loaded vertical jumps and sprint performance
Source: PLoS One. 2022 Sep 22;17(9):e0274962. doi: 10.1371/journal.pone.0274962 (PMC9499257; doi:10.1371/journal.pone.0274962)
Supplement: S2 Fig — (PDF) [file pone.0274962.s003.pdf]

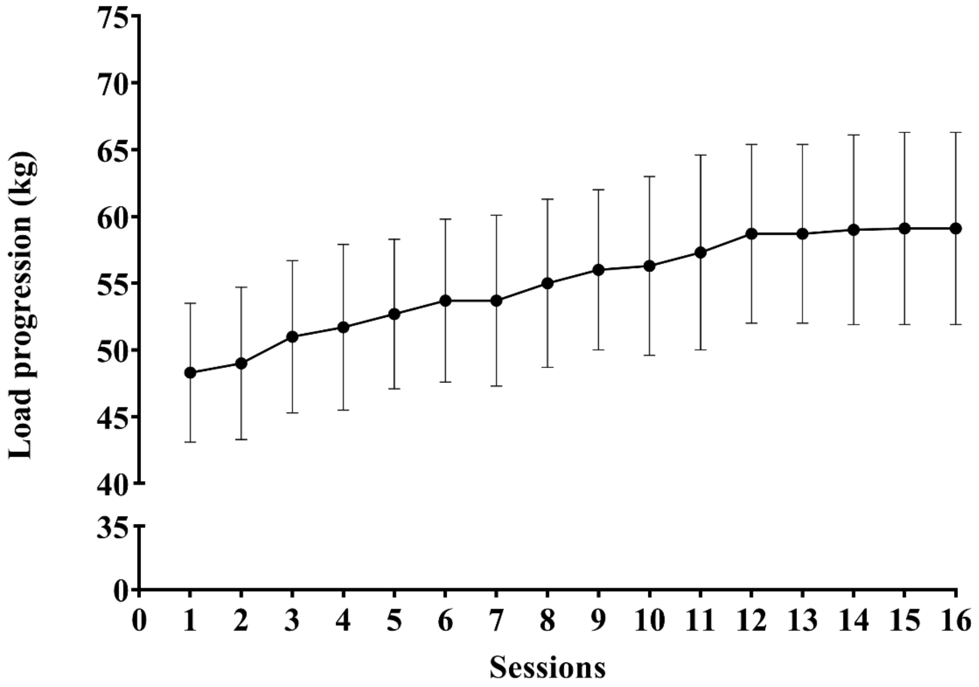

S3 supplemental material. The load progression in the WL group was represented by the adjustments performed in the power clean from the knee exercise.
